# The Demands of a Women’s College Soccer Season

**DOI:** 10.3390/sports6010016

**Published:** 2018-02-23

**Authors:** Jeremy A. Gentles, Christine L. Coniglio, Matthew M. Besemer, Joshua M. Morgan, Michael T. Mahnken

**Affiliations:** 1 Department of Sport, Exercise, Recreation, and Kinesiology, East Tennessee State University, Johnson City, TN 37614, USA; coniglio1918@gmail.com; 2 Department of Health Sciences, Armstrong State University, Savannah, GA 31419, USA; matt.besemer@cox.net (M.M.B.); josh2morgan@gmail.com (J.M.M.); mtmahnken@gmail.com (M.T.M.)

**Keywords:** soccer, accelerometer, Impulse Load, GPS, microsensor, Training Load, workload

## Abstract

The purpose of this study was to use GPS, accelerometers, and session rating of perceived exertion (sRPE) to examine the demands of a Division II women’s soccer team. Data was collected on 25 collegiate Division II women’s soccer players over an entire regular season (17 matches and 24 practices). Zephyr^TM^ BioHarnesses (BHs) were used to collect tri-axial acceleration information and GPS derived variables for all matches and practices. Acceleration data was used to calculate Impulse Load, a measure of mechanical load that includes only locomotor related accelerations. GPS was used to quantify total distance and distance in six speed zones. Internal Training Loads were assessed via sRPE. Mean Impulse Load, total distance, and sRPE during match play was 20,120 ± 8609 N·s, 5.48 ± 2.35 km, and 892.50 ± 358.50, respectively. Mean Impulse Load, total distance, and sRPE during practice was 12,410 ± 4067 N·s, 2.95 ± 0.95 km, and 143.30 ± 123.50, respectively. Several very large to nearly perfect correlations were found between Impulse Load and total distance (r = 0.95; *p* < 0.001), Impulse Load and sRPE (r = 0.84; *p* < 0.001), and total distance and sRPE (r = 0.82; *p* < 0.001). This study details the mechanical demands of Division II women’s soccer match play. This study also demonstrates that Impulse Load is a good indicator of total distance.

## 1. Introduction

The demands of women’s soccer match play have been detailed in a substantial body of literature [1]. Typical match play duration is 90 min, consisting of two 45 min halves, and one 15 min half-time. Average total distances covered per player during elite women’s soccer match play are 8500–10,300 m [2]. Soccer requires substantial aerobic and anaerobic fitness, with demands varying between competitions, by position, and match play style [1]. Aerobic fitness has been identified as an important fitness characteristic and it has been suggested that a VO_2_max of ~55 mL/min/kg is sufficient to play high level women’s soccer [2,3]. While the majority of match play is spent at velocity thresholds associated with walking and low speed running, average heart rate (HR) and peak HR in elite female match play have been reported to be 87% and 97% of max HR [1], respectively. In professional women’s soccer, it has been reported that mean sprint distance is ~15.1 ± 9.4 m, individual sprint time is ~2.3 ± 1.5 s, and time between sprints is ~2.5 ± 2.5 min [4]. The high mean match play HR and sprinting requirements illustrate the importance of anaerobic fitness for women’s soccer match play.

Recently, distance in velocity thresholds associated with high speed running (HSR) and sprinting has gained the attention of researchers [4,5]. Historically, definitions of HSR and sprinting were derived from velocity thresholds established from men’s soccer [5]. However, there is a growing body of literature focused on velocity thresholds that better define HSR and sprinting in women’s soccer [5]. For instance, HSR (19.8–25.1 kph) and sprinting thresholds (≥25.1 kph) used to describe speed zones of elite male match play seem to exaggerate the physical abilities of elite female players [5]. Previous research has underestimated the distance completed during HSR and sprinting in professional women’s soccer as a result of using velocity thresholds for male soccer players [6]. Although HSR and sprinting contribute relatively little to total distance during match play, to better quantify HSR and sprinting demands, profiles for female HSR and sprinting have been recently established. Based on the latest evidence, HSR and sprinting thresholds in collegiate and professional women’s soccer should be approximately 15 kph and >20 kph, respectively. Currently, the body of literature describing HSR and sprinting in women’s soccer using these recommended velocity thresholds is limited, particularly at the collegiate level [5]. As a result of these new recommendations, distance in HSR and sprinting should be further investigated at various levels of women’s soccer.

GPS is a common method used by sport science and coaching staff to quantify total distance and distance in speed zones for a variety of sports [7,8]. Although GPS is often used to quantify the demands of a variety of sports, as alluded to previously, few studies have used GPS to quantify the demands of women’s soccer, and fewer yet have assessed the demands of women’s college soccer [5]. The dearth of evidence detailing the demands of women’s college soccer as measured via GPS is noteworthy, as it is important to distinguish between the demands of women’s college soccer and women’s soccer more generally. Collegiate soccer allows for unlimited substitutions while international rules soccer allows only three substitutions per match, which may make it inappropriate for practitioners to design practice and conditioning sessions for college soccer based on evidence from soccer matches following international rules.

Despite the popularity of GPS use in team sport, practitioners should be aware of the potential limitations of GPS and the specifications of GPS units themselves. The circular error probability (CEP) of GPS has been reported to be approximately 1–5 m [9,10], meaning that 50% of an athlete’s recorded positions will be within 1–5 m of the athlete’s true position. The GPS chipset and environmental factors such as cloud cover and obstructions may impact GPS accuracy by influencing CEP, satellite acquisition times, and the number of satellites the GPS unit is able to connect with. It is generally agreed upon that GPS accuracy improves as distance increases, while GPS accuracy decreases as speed increases, particularly during HSR and sprinting [11,12,13,14]. In order to increase accuracy, it is suggested that GPS units should sample at 5–15 Hz [15,16], with some evidence recommending no less than 10 Hz and other suggestions that 10 Hz is superior to 15 Hz [13,17]. However, the discrepancy in sampling frequency recommendations may also be a result of different GPS chipsets and algorithms used to calculate distance and speeds between devices sampling at different rates [16]. The authors of this study are not aware of a single study that has compared GPS devices sampling at different rates (i.e., 1, 5, and 10 Hz) while also configured with the same chipset. Regardless of sampling frequency and algorithm/filter used, GPS will likely misrepresent position, distance, and speed due to accuracy limitations, particularly in sports that require repeated changes of direction, HSR, and sprinting.

Accelerometers and inertial measurement units (IMUs) have been used to quantify workloads in a variety of sports [18,19,20] and are most often measured with triaxial accelerometers and reported as the sum of acceleration in three movement planes (side-to-side; forwards and backwards; up and down). Accelerometers and GPS are frequently combined into the same unit, thus allowing GPS- and accelerometry-based workloads to complement one another. A strong relationship between total distance- and accelerometry-based workloads have been reported [18,21], although accelerometers provide potential benefits over GPS, as they measure all movement and can be used to quantify the number of accelerations and decelerations, number and magnitude of impacts, step and jump counts, and a variety of other variables [22]. Accelerometers also function indoors, do not require the use of satellites, and do not suffer from the same potential loss of signal that GPS does. Recently, Buchheit et al. [23] suggested that Force Load, an accelerometry-derived Training Load that includes only locomotor related events and impacts, may have numerous advantages compared to Training Loads obtained via GPS as well as other common accelerometry-based Training Loads that include all measured accelerations in their calculation. Currently, the most common accelerometry-based Training Load reported in the literature is Player Load [24]. Player Load includes all accelerations in the Training Load calculation and is calculated as the sum of absolute differences of acceleration divided by the sampling frequency of the device (generally a triaxial accelerometer sampling at 100 Hz) [25]; the formula for Player Load is shown below. However, Player Load may misrepresent Training Load, since activities of non-locomotor origin are included, and Player Load is the sum of absolute differences of acceleration and not the sum of the absolute values of acceleration. To our knowledge, not a single study has been published describing the accelerometry-derived Training Loads of women’s college soccer.

$$\mathrm{Player}\;\mathrm{Load}=\;\overset{n}{\underset{s=1}{\sum }}\frac{\sqrt{{\left(x_{s\;=\;i\;+\;1}-\;x_{s\;=\;i}\right)}^{2}+{\left(y_{s\;=\;i\;+\;1}-\;y_{s\;=\;i}\right)}^{2}\;+{\left(z_{s\;=\;i\;+\;1}-\;z_{s\;=\;i}\right)}^{2}}}{100}$$

There is currently limited GPS evidence and a complete absence of investigations addressing accelerometry-based workloads in women’s college soccer. Furthermore, in order to address the shortcomings of GPS and common accelerometry-derived Training Loads, it may be beneficial to use accelerometry-based Training Loads that include only locomotor-related events, as suggested by Buchheit et al. [23]. The purpose of this study was therefore twofold. First, in order to describe the demands of women’s college soccer, this study aimed to quantify the external and internal Training Loads of practices and matches throughout an entire regular season in women’s college soccer. External demands were quantified using GPS to calculate total distance and distance in speed zones, while accelerometers were used to measure locomotor-related events only. Internal Training Loads were quantified via session rating of perceived exertion (sRPE). Second, this investigation served to explore the relationships between accelerometry-derived Training Loads that include only locomotor activity, and Training Loads assessed using GPS and sRPE.

## 2. Methods

### 2.1. Subjects

Twenty-five collegiate DII women’s soccer players participated in the study (age 20.2 ± 1.1 years, height 166.3 ± 5.9 cm, weight 62.0 ± 7.0 kg, Yo-Yo Intermittent Recovery Test Level 1 Distance 1069 ± 255 m, countermovement vertical jump height 28.6 ± 3.4 cm). This investigation was approved by the Institutional Review Board and all participants completed and signed university-approved informed consent.

### 2.2. Operation of Wearable Device

Each athlete was assigned and familiarized with the wear and operation of the Zephyr^TM^ BioHarness (BH; Zephyr Technology Corporation, Annapolis, MD, USA) during preseason training. Each BH included a Biomodule (version 3) and strap. Athletes were equipped with a BH that was worn during competition. The BH strap was placed at the level of the xyphoid process and the Biomodule was positioned on the midaxillary line. The Biomodule contains a HR sensor and triaxial accelerometer that sample at 250 Hz and 100 Hz, respectively. Data from the HR sensor was not included in this study. BH data was downloaded to and analyzed with OmniSense^TM^ Analysis (version 4.1.4; Zephyr Technology Corporation, Annapolis, MD, USA). GPS units (BT-Q1300ST GPS, Qstarz International Co., Taipei, Taiwan) sampling at a true rate of 5 Hz (not interpolated) were also worn by each participant. Each GPS unit was attached to the BioHarness strap and interfaced with the Biomodule via Bluetooth.

The validity and reliability of BH-derived accelerometry loads has been demonstrated during a variety of tasks. Johnstone et al. [26] assessed the validity of the BH using a discontinuous incremental treadmill protocol and BH precision was evaluated via tilt table. During the treadmill protocol (0–12 kph), very strong relationships were demonstrated between BH-derived accelerometry loads and oxygen uptake (r = 0.97, *p* ≤ 0.01), as well as mean step count (r = 0.99, *p* ≤ 0.01). Using a tilt table, the BH was able to determine angle change when compared to a Flexometer (Leighton, Spokane, WA, USA); there was a very strong relationship between the BH and Flexometer (r = 0.99; *p* ≤ 0.01) and narrow limits of agreement between the measured angle of both devices (0.20 ± 2.62). Using the same treadmill and tilt table protocols used above [26], between subject, intra device, and inter device reliability has been demonstrated to be very strong with calculated intraclass correlation coefficient (ICC) and coefficient of variation (CV) consistently ≥0.99 and <8, respectively [27]. During a discontinuous field-based walk-jog-run protocol, there was a very strong relationship between accelerometry loads and oxygen uptake (r > 0.90; *p* < 0.01) and simultaneous wear of two BHs showed that inter device reliability was very strong (ICC = 0.93; CV = 10.3) [28]. Interestingly, during ten flights totaling 25,700 s in an F/A18 aircraft, the BH has also been shown to be a valid measure of gravitation force exhibiting very strong relationships (r = 0.92; r = 0.93) with the F/A-18’s Carrier Aircraft Inertial Navigation System-2 (CAINS-2) and ActiGraph WGT3X-BT Monitor (ActiGraph LLC, Pensacola, FL, USA), respectively [29].

The BT-Q1300ST GPS unit is equipped with the MediaTek II (MTKII) chipset and the accuracy of the MTKII chipset has been established on several occasions. The MTKII chipset has demonstrated greater accuracy (mean CEP = 5.0 m) and the lowest satellite acquisition times (mean time = 26.3 s) under a variety of environmental conditions when compared to other commercially available GPS units [10]. Wu et al. [30] showed that during static and dynamic conditions, the MTKII chipset had the lowest outdoor satellite acquisition times, the lowest percentage of signal loss, and the lowest outdoor horizontal dilution of precision when compared to six other GPS units. The reliability of the MTKII chipset has been shown to be very high to nearly perfect (ICC = 0.80–1.0) when quantifying distances in different speed zones (>19 kph to 0–5 kph) [31].

### 2.3. Match Time

Data was collected on 25 players over an entire regular season (17 matches and 24 practices). All BH and GPS units were powered on by the researchers prior to the start of each match and practice. Data collected in each of the 17 matches was categorized according to warm-up and match play segments. Warm-up included all activities prior to the beginning of match play. Warm-up activities included jogging, multidirectional running, dynamic stretching, jumping and bounding, change of direction, short sprints, and soccer-specific drills (dribbling, passing, tackling, shooting, etc.). Match play included two 45 min halves, a 15 min half-time, and overtime periods if they occurred; only three overtime periods occurred during the season. A regulation National Collegiate Athletic Association (NCAA) DII soccer match is 90 min, consisting of two- 5 min halves, one 15 min half-time break, and overtime periods are 10 min in duration if they are required. If stoppage time occurs, it is added to the end of standard play in each half and overtime. Total time played during match play was also recorded for each player. A total of 392 match and 522 practice sessions were analyzed. Due to erroneous GPS data such as zero distance recorded or excessive high peak speeds, some GPS-related data was removed for analysis. In total, accelerometry data was analyzed for all 392 match and 522 practice sessions. GPS data was analyzed from 305 match and 407 practice sessions.

### 2.4. Accelerometry

Gravitational forces (1 g = 9.81 m/s^2^) were recorded to describe acceleration data collected from BH at 100 Hz. Total mechanical loads were expressed as Impulse Load. Impulse Load is the accumulated mechanical load equal to the sum of areas under the 3-axis accelerometry curves and expressed as N·s. Impulse Load only includes detected locomotor events (e.g., walking, running, bounding, jumping) and impacts. Mean Impulse Loads were calculated for practice, warm-up, and match play. The formula for Impulse Load is displayed below, where *x* = g forces in the medio-lateral (“side-to-side”) plane, *y* = g forces in the anterio-posterior (“forwards and backwards”) plane, and *z* = g forces in the vertical (“up and down”) plane [32].

$$\mathrm{Impulse}\;\mathrm{Load}=\;\overset{n}{\underset{s=1}{\sum }}\frac{\sqrt{x_{s}^{2}+y_{s}^{2}+z_{s}^{2}}}{9.8067}$$

### 2.5. GPS

GPS units were used to collect total distance (km) and distance in speed zones during practice, warm-up, and match play. Speed was divided into six different zones: 1.0–4.99 kph (Zone 1); 5–9.99 kph (Zone 2); 10–14.99 kph (Zone 3); 15–19.99 kph (Zone 4); 20–24.99 kph (Zone 5); ≥25 kph and greater (Zone 6). All GPS units were turned on approximately 10 min prior to use to ensure satellite signals were acquired.

### 2.6. sRPE

Approximately 15 min after each practice and match, athletes reported their rating of perceived exertion (RPE) using the Borg CR-10 RPE scale [33]. Session rating of perceived exertion was calculated by multiplying the reported RPE times the session duration in minutes [33]. Practice session duration included the entire duration of the practice session while match session duration included total time between the start of warm-up through the end of match play.

### 2.7. Statistics

Data were analyzed with JASP (version 0.8.3.1) and expressed as means, standard deviations, and 95% confidence intervals (CIs). The relationships between Impulse Load and total distance, Impulse Load and sRPE, and total distance and sRPE were assessed using the Pearson product-moment correlation coefficient. The magnitudes of the correlations were interpreted via the following standard: trivial = 0.0–0.1; small = 0.1–0.3; moderate = 0.3–0.5; large = 0.5–0.7; very large 0.7–0.9; nearly perfect = 0.9–1.0 [34].

## 3. Results

Team mean warm-up duration was 45.64 ± 6.65 min and match mean duration was 116.72 ± 12.28 min. Mean Impulse Load, total distance, and sRPE during match play were 20,120 ± 8,609 N·s, 5.48 ± 2.35 km, and 892.50 ± 358.50, respectively. Mean Impulse Load, total distance, and sRPE during practice were 12,410 ± 4,067, 2.95 ± 0.95 km, and 143.30 ± 123.50, respectively. Table 1 provides team means and standard deviations for session duration and all GPS-, accelerometry-, and sRPE-derived metrics during practice, match warm-up, and match play.

**Figure 1:**
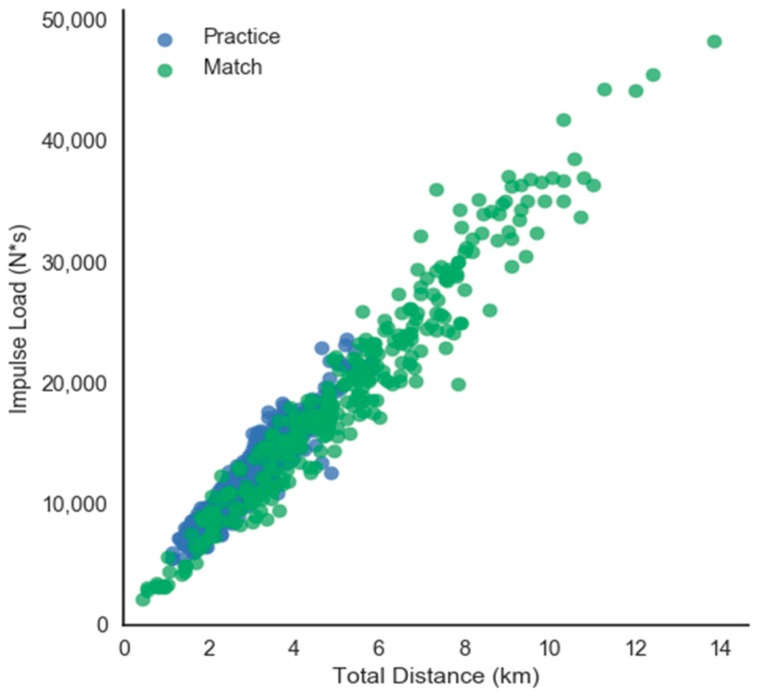
Impulse Load and total distance.

Several very large to nearly perfect correlations were found between Impulse Load and total distance (r = 0.95; *p* < 0.001), Impulse Load and sRPE (r = 0.84; *p* < 0.001), and total distance and sRPE (r = 0.82; *p* < 0.001). Figure 1 illustrates the relationship between Impulse Load and total distance.

## 4. Discussion

**Figure 2:**
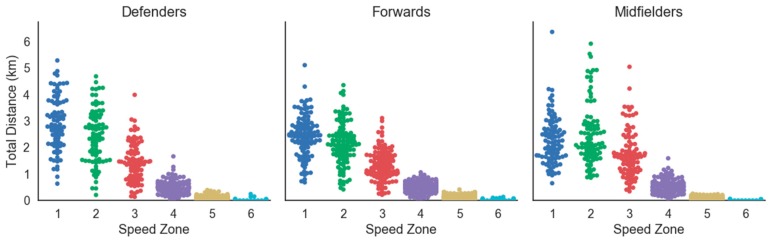
Swarm plot of distance in all speed zones by player position during match play.

**Figure 3:**
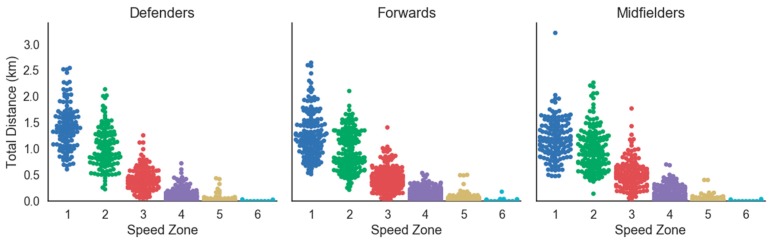
Swarm plot of distance in all speed zones by player position during practice.

To our knowledge, this was the first study to assess accelerometry-based workloads in women’s college soccer as well as the first study to detail the demands of Division II women’s soccer match play. Previous research has investigated distance in speed zones and total distances. However, most of this research has focused on professional and national team levels, with limited research in women’s college soccer [5]. Collegiate soccer allows for unlimited substitutions, which differs from rules of professional and national team matches that only permit thee substitutions per match. One reason provided for the allowance of unlimited substitutions is due to the frequency of match play in college soccer. NCAA soccer teams generally play twice a week, and professional soccer teams often compete once a week [5]. As a result of different substitution rules, most starting players at the professional level must play the entire match, while college starting players have no such requirement. This difference in substitution patterns make it difficult to compare physiological and mechanical workloads among high level and collegiate play. Unlimited substitutions allow for large variances in match-to-match total distance, distance in speed zones, and total Impulse Load accumulated by each athlete. To demonstrate this, during match play (MP), mean time played was 45.32 ± 26.01 min, mean Impulse Load was 20,120 ± 8609 N·s, and mean total distance was 5.48 ± 2.35 km. Time played during match play ranged from 1.0–110.0 min, the highest and lowest Impulse Load during match play was 48,280 N·s and 4215 N·s, respectively, and total distance ranged from 1.09–13.85 km. Furthermore, Figure 2 and Figure 3 present swarm plots illustrating the distribution of distance in all speed zones by player position during matches and practice, respectively. This variability should be considered when quantifying the demands of women’s collegiate soccer.

Previous research in women’s soccer has categorized speed zones based on gender-specific abilities and locomotor activity that is associated with those speed zones [5,35]. Recently updated definitions of walking and running activities in women’s soccer are presented in three categories; (1) standing/walking/low speed running (<12 kph); (2) moderate/high speed running (12.1–20 kph), and (3) sprinting (>20 kph) [5,35]. Table 1 presents time in speed zones from this study according to six speed zones. The results of this study, similar to findings of previous investigations in collegiate and professional women’s soccer [5], show that the majority of total match play distance occurs in speed zones that represent standing, walking, and low speed running. As detailed in Table 1, high speed running and sprinting contribute very little to total distance, although both have been identified as variables important for match play performance and injury [36].

Unsurprisingly, very large correlations were found between sRPE and external Training Loads (Impulse Load and GPS). Strong relationships between sRPE and both GPS- and accelerometry-derived training have been reported previously [37,38,39,40,41]. Perhaps more important is the relationship between Impulse Load and total distance (r = 0.95; *p* < 0.001) found in this study. While previous research has found a strong to very strong relationship between Player Load and total distance in soccer [21,41], the relationship found between Impulse Load and total distance seems to be uniquely strong. This may be because Impulse Load only includes locomotor activity and represents the sum of locomotor events. Accelerometry-derived Training Loads such as Impulse Load and Force Load may provide benefits over other accelerometry-based workloads such as Player Load [42], which includes all accelerations of the torso, including non-locomotor activities, and represents the sum of absolute differences of acceleration. The benefits of using accelerometry workloads that only include locomotor activity have been recognized previously [23], but limited examples currently exist in the literature related to workloads in sport [19].

**Figure 4:**
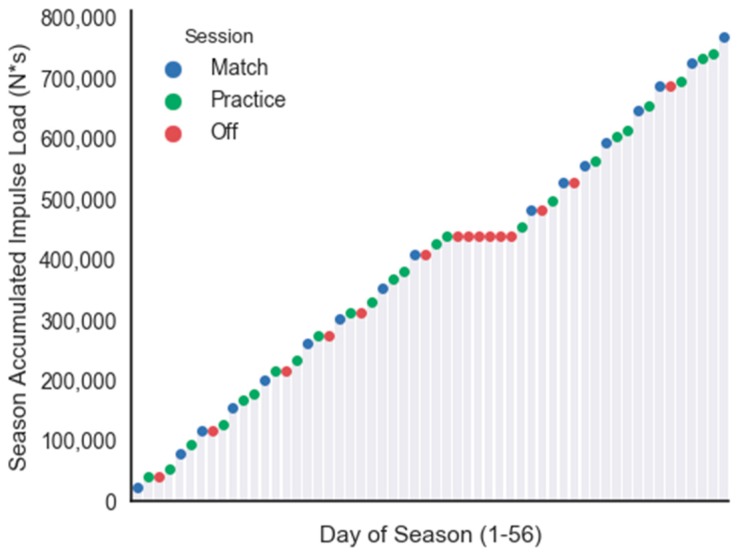
Team mean accumulated Impulse Load throughout the 56-day regular season.

Unique to this study was that Hurricane Matthew struck the southeast coast of the United States during the middle of the regular season. Due to this major hurricane, a mandatory evacuation was issued that resulted in multiple match postponements and missed practices. Matches missed during this period were made up during the last two weeks of the regular season. Figure 4 displays the frequency of practices, matches, and days off, as well as the team mean accumulated Impulse Load throughout the 56-day regular season. No contact with the team occurred during the mandatory evacuation period (days 31–36). It can be seen that the frequency of matches and the rate of accumulating Impulse Load both increased during the second half of the regular season (days 37–56). During the first thirty days of the season, nine matches were played, with two to three days between matches. During the last 20 days of the season, eight matches were played, with only one to two days between matches. While injury data was not presented in this study, it is reasonable to assume that this situation may have resulted in higher levels of fatigue that may have negatively influenced injury risk and performance.

There are several important limitations that should be considered in this study. First, GPS in general becomes less accurate as speed increases and with repeated change of direction. Therefore, distances in speed zones >20 kph, as well as speed and distance associated with changing direction, may be misrepresented. Second, while the literature has not clearly assessed the potential benefits of using GPS units that sample at >5 Hz, it is possible that the GPS units used in this study, which sample at 5 Hz, are not able to capture positional information frequently enough to accurately measure distance during HSR and sprinting. Third, the algorithms used to identify the locomotor events that are included in Impulse Load are proprietary. As a result, it is difficult to verify with absolute certainty that only locomotor events are being included in the calculation of Impulse Load.

## 5. Practical Applications and Future Research

Coaches and sport scientists can use Impulse Load, total distance, distance in speed zones, and sRPE to help guide and design effective practices and training programs that prepare athletes for the demands of women’s college soccer. However, to ease the management of what can be a large volume of data, practitioners may benefit from limiting the number of metrics used to quantify the demands of their athletes and sports. Considering the results of this study and the recommendation of Buchheit et al. [23], accelerometry-derived Training Loads such as Impulse Load and Force load, that include only locomotor activities, may provide benefits over GPS- and other accelerometry-based Training Loads. Additionally, accelerometers do not suffer from the same limitations as GPS, such as signal loss due to inherent limitations of the device and environmental conditions. Accelerometers also sample at higher frequencies and battery life is also generally superior to GPS.

This research also illustrates the varied MP demands that result from unlimited substitutions in women’s college soccer. Practitioners responsible for the development of women’s college soccer athletes should consider that the demands of women’s college soccer may differ dramatically from women’s soccer that follows international rules; this is largely due to the fact that collegiate soccer allows for unlimited substitutions, while professional and national team soccer only permit three substitutions per match. Considering the substitution rules of women’s college soccer, practitioners should quantify the MP demands of their specific team and athletes in order to design practices and conditioning activities that suit the strategies employed by the sport coach.

Future research should assess whether Impulse Load and its locomotor components (e.g., stepping events) are related to variables acquired by GPS, such as distance in speed zones. Additionally, Impulse Load should be used to quantify the Training Loads for individual soccer drills and other practice elements. Practitioners would also benefit from research that develops non-proprietary algorithms for locomotor event detection and quantification, as well as additional investigations to assess the impact of sampling rates on GPS accuracy.

## 6. Conclusions

This study was the first to quantify the seasonal demands of women’s college soccer using sRPE, GPS, and Impulse Load, an accelerometry-derived Training Load that includes only locomotor activities. Large variations in total distance, distance in speed zones, Impulse Load, and sRPE were found during MP; this was largely due to the number of substitutions. However, consistent with previous investigations, a relatively small portion of total distance consists of HSR and sprinting. This study has also detailed the relationship between Impulse Load, total distance, and sRPE. Due to the nearly perfect relationship between Impulse Load and total distance, Impulse Load may be used as a proxy for total distance.

## Figures and Tables

**Table 1:** Team mean session duration and all GPS-, accelerometry-, and sRPE-derived metrics during practice, match warm-up, and match play. RPE: rating of perceived exertion; sRPE: session rating of perceived exertion.

	Practice	Match Warm-Up	Match Play (Played Only)
Session Duration (min)	75.77 (±16.65)	46.22 (±6.68)	116.50 (±12.12)
Duration Played (min)	NA	NA	45.32 (±26.01)
Distance in Speed Zone 1 (km)	1.30 (±0.44)	1.04 (±0.23)	1.74 (±0.79)
Distance in Speed Zone 2 (km)	1.00 (±0.40)	0.70 (±0.23)	1.83 (±0.92)
Distance in Speed Zone 3 (km)	0.47 (±0.25)	0.26 (±0.11)	1.32 (±0.73)
Distance in Speed Zone 4 (km)	0.15 (±0.13)	0.09 (±0.05)	0.46 (±0.25)
Distance in Speed Zone 5 (km)	0.03 (±0.07)	0.01 (±0.02)	0.11 (±0.08)
Distance in Speed Zone 6 (km)	0.00 (±0.01)	0.00 (±0.01)	0.02 (±0.02)
Total Distance (km)	2.95 (±0.95)	2.1 (±0.47)	5.48 (±2.35)
Impulse Load (N·s)	12,410 (±4067)	9694 (±1902)	20,120 (±8609)
Session RPE	143.30 (±123.50)	NA	892.50 (±358.50)
